# Improved blastocyst development of single cow OPU-derived presumptive zygotes by group culture with agarose-embedded helper embryos

**DOI:** 10.1186/1477-7827-9-121

**Published:** 2011-08-24

**Authors:** Gautam Kumar Deb, Jong In Jin, Tae Hyun Kwon, Byung Hyun Choi, Jae Il Bang, Shukla Rani Dey, In Rae Cho, Il Keun Kong

**Affiliations:** 1 Division of Applied Life Science (BK21 program), Graduate School of Gyeongsang National University, Jinju 660-701, Republic of Korea; 2 Central Branch of Gyeongnam Livestock Promotion Research Institute, Kimhae 621-833, Republic of Korea; 3 Institute of Agriculture and Life Science, Gyeongsang National University, Jinju 660-701, Republic of Korea

## Abstract

**Background:**

The *in vitro *culture of presumed zygotes derived from single cow ovum pick-up (OPU) is important for the production of quality blastocysts maintaining pedigree. The aim of the present study was to evaluate the agar chip-embedded helper embryo coculture system for single cow OPU-derived zygotes by assessing embryo quality.

**Methods:**

Cumulus oocyte complexes (COCs) were collected from Hanwoo cows with high genetic merit twice a week using the ultra-sound guided OPU technique and from slaughterhouse ovaries. The Hanwoo cow COCs and slaughterhouse ovaries were matured *in vitro*, fertilized *in vitro *with thawed Hanwoo sperm and cultured for 24 h. The presumed zygotes were subsequently placed in three different culture systems: (1) control OPU (controlOPU) with single cow OPU-derived presumed zygotes (2~8); (2) agar chip-embedded slaughterhouse helper embryo coculture (agarOPU) with ten presumed zygotes including all presumed zygotes from a cow (2~8) and the rest from agar chip-embedded slaughterhouse presumed zygotes (8~2); and (3) slaughterhouse *in vitro *embryo production (sIVP) with ten slaughterhouse ovary-derived presumed zygotes, each in 50 μL droplets. Day 8 blastocysts were assayed for apoptosis and gene expression using real time PCR.

**Results:**

The coculture system promoted higher blastocyst development in OPU zygotes compared to control OPU zygotes cultured alone (35.2 vs. 13.9%; P < 0.01). Genes predicted to be involved in implantation failure and/or embryo resorption were down-regulated (P < 0.05) in control OPU zygotes (*CD9*, 0.4-fold; *AKRAB*1, 0.3-fold) and in cocultured zygotes (*CD9*, 0.3-fold; *AKRAB*1, 0.3-fold) compared to sIVP blastocysts (1.0-fold). Moreover, genes involved in implantation and/or normal calf delivery were up-regulated (P < 0.05 to P < 0.01) in control OPU zygotes (*PGSH*2, 5.0-fold; *TXN*, 4.3-fold; *PLAU*, 1.7-fold) and cocultured zygotes (*PGSH*2, 14.5-fold; *TXN*, 3.2-fold; *PLAU*, 6.8-fold) compared to sIVP (1.0-fold) blastocysts. However, the expression of *PLAC8, TGF-β1, ODC1*, *ATP5A1 *and *CASP3 *did not differ between the three culture groups.

**Conclusions:**

Results show that the agar chip-embedded helper embryo coculture system enhances developmental competence and embryo quality in cultures of limited numbers of high pedigree single cow OPU presumed zygotes.

## Background

Ultrasound-guided transvaginal ovum pick-up (OPU) in combination with conventional *in vitro *fertilization (IVF) has enabled the production of large numbers of embryos from high genetic merit donors of different ages and physiological conditions [1-3]. Therefore, the OPU technique combined with IVF has the potential to improve genetic progress through the maternal lineage [4]. The efficiency of OPU-based *in vitro *embryo production (IVP) ranges between 11% in untreated cows to 30% in hormone-treated cows [2,5-8]. The number of COCs collected from a single cow during an OPU session varies from < 3 in non-stimulated cows [5] to > 9 in hormone-stimulated cows [8]. Approximately 50% of all OPU-derived COCs are of poor quality [5,8]. A culture system promoting consistent high blastocyst development and maintaining pedigree would be crucial to cattle breeding programs.

A minimal number of presumed bovine zygotes must be cultured within a droplet in order to maximize embryo developmental competence and quality by maintaining appropriate autocrine and paracrine signaling [9,10]. In a recent study, Senatore et al. [11] showed that agar-embedded helper embryos can improve the developmental competence of OPU oocytes. This novel finding suggested that agar-embedded slaughterhouse ovary-derived presumed zygotes may be cultured with small numbers of single cow OPU presumed zygotes to improve OPU embryo development and quality. However, the quality of the embryos derived by this technique remains to be evaluated. Culture conditions can modulate the expression of genes that affect embryo quality [12-17]. The quality of pre-attached blastocysts is usually predicted from the total number of blastomeres, the proportion of inner cell mass and the percentage of apoptotic blastomeres [18]. However, these criteria do not always ensure high calf output after embryo transfer. Improvements in quantitative real time PCR enabled the identification of several molecular markers of pre-implantation embryo quality, including genes involved in metabolic activity, stress response and reprogramming [19]. The expression pattern of thioredoxin (*TXN)*, placenta-specific 8 (*PLAC*8), prostaglandin G/H synthase-2 (*PGSH*2), urokinase-type plasminogen activators (*PLAU*), ornithine decarboxylase (*ODC1*), the alpha subunit of ATP synthase isoform 5 A1 *(ATP5A1*), aldo-keto reductase family 1 member B1 (*AKR1B*1) and CD9 (*CD*9) in bovine blastocysts reliably predicts quality in terms of implantation, early embryo loss/resorption and normal calf delivery [20]. Transforming growth factor beta 1(*TGF-β1*) was identified at the fetal-maternal interface and plays crucial roles during the post-transfer development of embryos [21].

The aim of the present study was to determine whether agar chip-embedded slaughterhouse-derived helper embryos may enhance the developmental competence and quality of single cow OPU-derived presumed zygotes in coculture.

## Methods

### Reagents

Unless otherwise indicated, all chemicals and media were purchased from Sigma Chemical Co. (St. Louis, MO, USA).

### Experimental design

**Figure 1:**
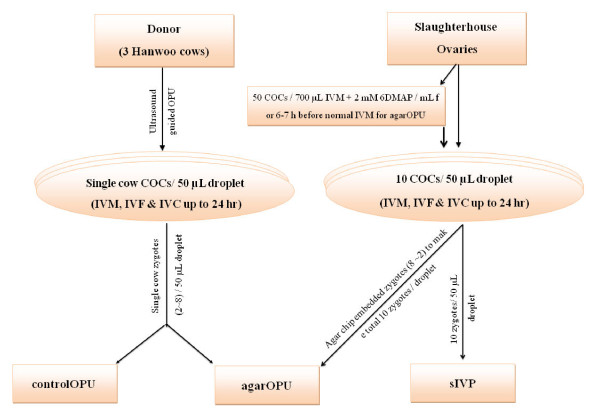
**Schematic representation of experimental design**. During an OPU session, all COCs retrieved from a single cow were subjected to *in vitro *maturation (IVM), *in vitro *fertilization (IVF) and *in vitro *culture (IVC-I) for up to 24 h. After 24 h of IVC, the presumed OPU zygotes were cultured either (1) alone (2~8) or (2) cocultured (2~8) with agar chip-embedded slaughterhouse presumed zygotes (8~2). Moreover, a group of ten slaughterhouse-derived presumed zygotes were cultured (sIVP) to supply presumed zygotes for coculturing with OPU presumed zygotes or for the evaluation of embryonic development.

Three different culture systems were tested to evaluate embryo development in OPU and slaughterhouse presumed zygotes including: (1) "control OPU (controlOPU)" with single cow OPU derived presumed zygotes (2~8 depending on availability), (2) "agar chip embedded slaughterhouse helper embryo coculture (agarOPU)" with a threshold of total ten presumed zygotes including all presumed zygotes from a donor cow (2~8 depending on availability) and the rest from agar chip embedded slaughterhouse presumed zygotes (8~2 depending on OPU presumed zygotes) and (3) "slaughterhouse IVP control (sIVP)" with ten presumed slaughterhouse derived zygotes each in 50 μL droplets of IVC medium. *In vitro *maturation, IVF and IVC (up to 24 h) were performed with single cow OPU COCs, each in 50 μL droplets. A detailed experimental design is presented in Figure 1. The COCs of alternative OPU sessions were used either in control OPU cultures (controlOPU) or in cocultures (agarOPU). The slaughterhouse COCs were pre-treated with 2 mM 6-dimethylaminopurine (DMAP) for 6**~**7 h to generate agar chip-embedded helper embryos or used for continued *in vitro *culture (dmapIVP) to evaluate developmental rates and embryo quality in response to DMAP.

### Oocyte donors

Two sets of four- to six-year-old Hanwoo cows (three cows per set) of high pedigree were used as oocyte donors. The cows were housed in a barn and fed commercially available concentrate pellets and straw, with fresh water provided *ad libitum*. Experimental cows were housed together in an open stall, in the same environmental conditions. The experimental cows had functional ovaries during the experimental period, as confirmed by transrectal palpation. The experimental procedures were approved by the Gyeongsang National University Association for the Accreditation of Laboratory Animal Care. The first set of donor cows was used for 22 OPU sessions and the second set for eight sessions.

### Ovum pick-up

A twice/week OPU schedule was followed throughout the experimental period, without subjecting the cows to hormonal stimulation. Follicles were visualized using an ultrasound scanner equipped with a 6.5 Mhz sectorial probe (Madison, WI, USA) fitted in a custom made intra-vaginal OPU probe-holder. An 18 gauge disposable hypodermic needle (Cook Veterinary Products, Queensland, Australia) connected to a 50 mL conical tube (BD Falcon, NJ, USA) by Teflon tubing (Cook Veterinary Products) was used for follicular puncture. Negative pressure was applied using a vacuum aspiration pump (GAST, MI, USA) and the aspiration vacuum was adjusted to a flow rate of 15 mL of water per minute. The COC collection tube and aspiration medium were kept at 38°C in a water bath. Oocytes were collected in Tyrodes lactate (TL)-Hepes medium enriched with 2% (v/v) fetal calf serum, 100 iu/mL penicillin, 0.1 mg/mL streptomycin and 5 iu/mL heparin. To minimize abdominal straining during OPU, epidural anesthesia was performed with 5 mL of lidocaine (Je-Il Pharmaceutical, Daegu, Republic of Korea). Follicles ≥3 mm in diameter were punctured during each OPU session.

### *In vitro *maturation (IVM)

The follicular fluid collected in TL-Hepes in 50 mL conical tubes was decanted into an EM-CON embryo filter (Agtech Inc., Spring Valley, WI, USA) and the oocytes were isolated under a stereomicroscope. The oocyte recovery rate was calculated as the number of oocytes recovered and expressed as the percentage of the total follicles aspirated for each cow. The oocytes were divided into four groups according to the presence or absence of cumulus cells surrounding the oocytes and their cytoplasmic configuration [22]. All COCs from a single cow were washed in TL-Hepes before a final wash in maturation medium and transferred into 50 μL droplets of IVM medium (TCM199 supplemented with 10% (v/v) fetal bovine serum (FBS), 1 μg/mL β-estradiol, 10 μg/mL FSH, 0.6 mM cystein and 0.2 mM Na-pyruvate) for 22~24 h under mineral oil.

### *In vitro *fertilization (IVF)

Frozen-thawed Hanwoo spermatozoa from a single batch of semen were used throughout the study. Sperm preparation procedures were described previously [23]. Briefly, thawed sperm was washed with Dulbecco's PBS and incubated with 20 μg/mL heparin sodium salt in IVF medium (Tyrode's lactate solution supplemented with 6 mg/mL fatty acid-free bovine serum albumin (BSA), 22 μg/mL sodium pyruvate, 100 iu/mL penicillin and 0.1 mg/mL streptomycin) for 15 min. The presumptive capacitated spermatozoa were finally diluted to 1 × 10^6 ^spermatozoa/mL of IVF medium. The matured COCs were cocultured with the presumptive capacitated sperm in a 50 μL droplet of IVF medium for 18~20 h.

### *In vitro *culture (IVC)

Eighteen to twenty hours after fertilization the cumulus cells surrounding the zygotes were denuded by pipetting in TL-Hepes medium in a 35 mm culture dish (SPL Life Sciences, Gyeonggi-do, Republic of Korea) with finely stretched pasteur pipettes. Presumed zygotes from individual cows (minimum two per cow during an OPU session) were then transferred into 50 μL droplets of modified CR1-aa [24] supplemented with 44 μg/mL Na-pyruvate, 14.6 μg/mL glutamine, 100 iu/mL penicillin, 0.1 mg/mL streptomycin, 3 mg/mL BSA and 310 μg/mL glutathione (IVC-I medium) for 24 h, and then cultured in medium in which the BSA had been replaced with 10% (v/v) FBS (IVC-II medium) until day 8 (day 0: day of IVF). BSA was replaced with FBS earlier than in a conventional CR1aa-based IVC system because it was difficult to handle agar-embedded embryos once they had been transferred to the IVC medium. Fifty percent of the medium was changed with fresh IVC-II medium at day 6.

Droplets of medium covered with mineral oil (M-8410) were pre-incubated for at least 2 h at 38.5°C in an atmosphere of 5% CO_2 _in humidified air. Cleavages and blastocyst development rates were evaluated as the proportion of OPU-derived presumed zygotes at day 3 and day 7 post-insemination, respectively. Day 8 blastocysts from the three donor cows were pooled and washed three times in PBS before being snap frozen in liquid nitrogen and stored at -80°C until RNA extraction or fixed in 4% (v/v) paraformaldehyde in PBS and kept at 4°C until the detection of blastomeres.

### Synchronization of slaughterhouse oocytes

The slaughterhouse COCs were aspirated as described previously [23]. Only grade 1 and 2 oocytes were used for subsequent IVM/IVF/IVC. To coordinate the culture schedule of OPU-derived COCs, the maturation of slaughterhouse COCs was suppressed by incubation with 2 mM DMAP in IVM medium for 6~7 h (50 COCs/700 μL) before the COCs were washed in IVM medium and then transferred into the final IVM droplets (10 COCs/50 μL droplet). The IVF and IVC procedures were the same as those described above for OPU oocytes.

### Preparation of agar-embedded helper embryos

**Figure 2:**
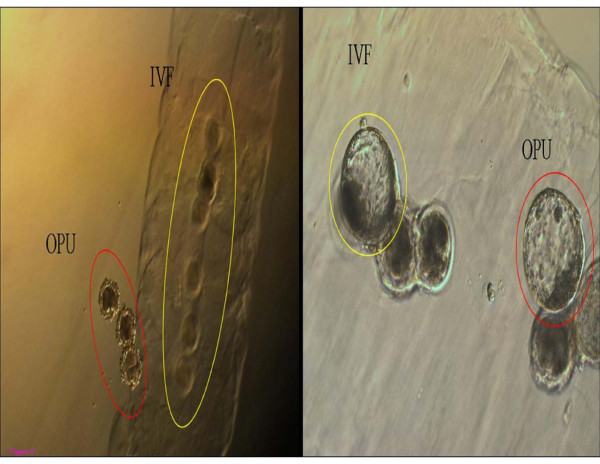
**Agar chip-embedded slaughterhouse helper embryo coculture for ovum pick-up (OPU) presumed zygotes**. IVF: slaughterhouse *in vitro *embryo production system. Ovals show OPU (red) and agar chip-embedded IVF (yellow) zygotes in a 50 μL droplet. Circles represent blastocysts developed from OPU (red) or IVF (yellow) zygotes.

Agar-embedded helper embryos were prepared according to Senatore et al. [11] with modification. Briefly, one gram of agarose (Type VII A9045) was added to 100 mL D-PBS and melted on a portable lab stove. Once melted, the agarose solution was aliquoted into ten 10-mL conical tubes (BD Falcon) and stored at 4°C until use. Before use, the agarose was again melted by heating at 60**°**C and was placed in an incubator set at 36~38 **°**C. The solution was poured into the wells of a 4-well dish (SPL Life Sciences) and the slaughterhouse zygotes were washed four to five times in the agar solution using a pasteur pipette with an opening a little larger than the diameter of an embryo. Once the agarose surrounding the zygotes solidified, the agar-embedded zygotes were transferred into wells already containing the OPU zygotes in culture medium (Figure 2).

To avoid possible disease transmission to OPU blastocysts from slaughterhouse embryos, ovaries were collected from physically healthy cows and transferred to the lab in separate flasks from those commonly used for IVP. Ovaries bearing blood-filled follicles were not used for COC aspiration. Before aspiration, the ovaries were washed several times in physiological saline. The aspirated COCs were washed two to three times in TL-Hepes containing antibiotic (100 iu/mL penicillin and 0.1 mg/mL streptomycin) and underwent a final wash in maturation medium. Moreover, slaughterhouse presumed zygotes were washed in IVC-I medium four to five times before being embedded in agar chips.

### Terminal deoxynucleotidyl transferase dUTP nick end labeling (TUNEL) staining

The number of blastomeres and the percentage of apoptotic blastomeres in day 8 blastocysts were detected by TUNEL staining using the In Situ Cell Death Detection Kit (Fluorescein; Roche Diagnostics Corp.; Indianapolis, IN, USA) as described previously [25]. Briefly, fixed embryos were washed twice with 0.3% (w/v) polyvinylpyrrolidone (PVP) in 1 M PBS (PVP-PBS) before being incubated in 0.5% (v/v) Triton X-100 in 0.1% (w/v) sodium citrate for 30 min at room temperature. After permeabilization, the embryos were washed twice in PVP-PBS and incubated in the dark in fluorescence-conjugated dUTP and terminal deoxynucleotide transferase for 1 h at room temperature. These TUNEL-stained embryos were then washed in PVP-PBS and incubated in PVP-PBS containing 10 μg/mL Hoechst 33342 for 10 min. Washing was then performed twice in PVP-PBS to remove excess Hoechst 33342 and the blastocysts were mounted on glass slides under cover slips to evaluate their nuclear configuration. The number of blastomeres in each blastocyst was counted under an epifluorescence microscope equipped with a mercury lamp. TUNEL-positive cells displayed bright red fluorescence. The total number of blastomeres was determined by the extent of green/blue fluorescence. The number of blastocysts subjected to TUNEL staining was seven for the controlOPU group, ten for the agarOPU group, 11 for the sIVP group and 20 for the dmapIVP group. Blastocysts of controlOPU (7), agarOPU (10) and sIVP (10) stained with 10 μg/mL Hoechst 33342 for 10 min during preliminary experiments were also included in the calculation of total blastomere numbers.

### Gene expression analysis

Messenger RNA (mRNA) was extracted from three sets (two to three day-8 blastocysts per set) of blastocyst samples for each group (controlOPU, agarOPU and sIVP) using Dynabeads mRNA Direct Kit (Dynal AS, Oslo, Norway) according to Deb et al. [25]. The mRNA samples were reverse transcribed in a final volume of 20 μL using iScript cDNA Synthesis Kit (Bio-Rad Laboratories, Hercules, CA, USA) according to the manufacturers' instructions. The cDNA was diluted by adding 30 μL RNase-free water per embryo equivalent. The limited yields of mRNA prevented any assessment of RNA integrity.

The primers were designed and tested for specificity using the Primer Designing Tool [26] and are shown in Table 1. qPCR was performed in duplicate in a CFX98 instrument (Bio-Rad Laboratories) using a 10 μL reaction mixture containing 0.2 mM of each bovine-specific primer, 1 × iQ SYBR Green Supermix (iQ SYBR Green Supermix kit, Bio-Rad Laboratories) and 1.0 μL of diluted cDNA. The cycling conditions were as follows: 95°C for 3 min followed by 44 cycles of 15 s at 95°C, 20 s at 57°C and 30 s at 72°C, and a final extension of 5 min at 72°C. Amplification was followed by a melting curve analysis using progressive denaturation during which the temperature was raised from 65 to 95°C at a transition rate of 0.5°C per second. A negative control (NTC) was performed for each gene. The target genes were quantified by the ΔΔ c(t) method using CFX manager V1.1 software (Bio-Rad Laboratories). Normalization was performed against two reference genes selected among four (*ACTB, 18s rRNA, GAPDH *and *YWHAZ) *that were tested with the target genes (*CD9, AKR1B1, PGSH2, TXN, PLAU, PLAC8, ODC1, TGF-B1, ATP5A1*, and *CASP3*). The average CV for the reference genes was 0.2286. The average M-value was 0.0816 for the blastocyst samples. The low M-value indicates the stability of the reference genes. PCR efficiency was calculated for each gene using relative calibration curves prepared from bovine uterine cDNA using a 10-fold dilution series in five points. The standard curves yielded correlation coefficients greater than 0.98 and reaction efficiencies between 90 and 110%. The qPCR was performed once with each three biological samples and at least one time with pooled cDNA from the three biological samples. The mean of four replicates was used for statistical analysis.

**Table 1:** Primers used for quantitative real-time PCR analysis

Gene name	GeneBank accession number	Forward primer (5'-3')	Reverse primer (5'-3')	Product length (base pairs)
*AKR1B1*	BT021058	TGCAACCCAAATACTCTTTT	AAAAGCCTAGCTGAAAGGAT	106
*ATP5A1*	M_174684.2N	GACCGATGGAAAGATCTCAG	AACCACCAAGCAACATGG	212
*CD9*	NM_173900	AGATCTTCCGAAGCAAATTC	CAAAGTTAGTGGCAAAGGAA	238
*ODC1*	NM_174130.2	CTATGTGATGTCAGGGCCAA	CACGTTAATACGCGTGGAAG	166
*PGSH2*	NM_174445	ATCCTCCCACAGTCAAAGAT	GCACATCACACACTCTGTTG	162
*PLAC8*	NM_001025325	TCTGACATTTTTACCGCTCT	ATTTCATTGCAGCATTTTCT	131
*PLAU*	NM_174147.2	ATCCCTCTGACTATCGCTAC	CAGGAATCTGTTTCCCACTG	151
*TGFβ1*	NM_001166068.1	TGGAGCTGTACCAGAAATATAGCAA	GCCACTGCCGCACAACTC	120
*TXN*	AF104105	TGATCAAGCCTTTCTTTCAT	TAATGGTGGCTTCAAGTTTT	195

*AKR1B1*: aldo-keto reductase family 1, member B1; *ATP5A1*: alpha subunit ATP synthase isoform 5 A1; *CD9*: CD9 molecule; *ODC1*: ornithine decarboxylase; *PLAU*: urokinase-type plasminogen activators; *PGSH2*: prostaglandin G/H synthase-2; *PLAC8*: placenta-specific 8; TGFβ1: transforming growth factor *beta 1*; *TXN*: thioredoxin.

Primer sequences fo*r *18s ribosomal RNA *(18srRNA)*, beta actin *(ACTB)*, caspase3 *(CASP3)*, glyceraldehyde-3-phosphate dehydrogenase *(GAPDH) *and tyrosine 3-monooxygenase/tryptophan 5-monooxygenase activation protein, zeta polypeptide *(YWHAZ) *were published previously [25].

### Statistical analysis

Data were analyzed using one way analysis of variance (ANOVA) and Duncan's Multiple Comparison Test using the SPSS program (Version 11.5; SPSS Inc., Chicago, IL, USA). However, the chi-square test was used to analyze oocyte recovery rate, percent of high quality oocytes (grade 1+2), cleavage and blastocyst development rate. Differences were considered significant at the 5% level unless otherwise indicated. Data were expressed as mean ± standard deviation.

## Results

### Follicle and oocyte dynamics

A total of 578 follicles were punctured during 30 OPU sessions from two sets of three cows (22 sessions for the first set and eight sessions for the second set), yielding 400 oocytes. The number of follicles punctured and of oocytes recovered per Hanwoo cow during an OPU session was 6.4 ± 2.1 (with a range of 2 to 10) and 4.4 ± 1.8 (with a range of 2 to 8), respectively. The oocyte recovery rate was 69.3%, of which 49.2% was of high quality (grade 1 and 2).

### *In vitro *embryo development efficiency

Development rates up to the cleavage stage did not differ between the controlOPU group and the agarOPU group; however, blastocyst development rates were higher in the agarOPU group than in the controlOPU group (P < 0.01). The cleavage and blastocyst development efficiency of the agarOPU group was similar to that of the sIVP culture system (Table 2). Moreover, the exposure of immature slaughterhouse COCs to DMAP for 6~7 h before IVM did not affect the cleavage and blastocyst development rates (Table 2).

**Table 2:** *In vitro *development potential and quality of bovine presumed zygotes

Culture system	Presumed zygote (n)	% cleaved (range in %)	% blastocyst (range in %)	Total blastomeres (range)	% apoptotic blastomeres (range in %)
ControlOPU	201	64.3^a ^± 32.0 (0-100)	13.9^a ^± 18.1 (0-57.1)	118.3^a ^± 24.4 (65-163)	4.6^a ^± 2.0 (1.3-7.5)
Agar OPU^x^	151	82.8^a^± 26.1 (0-100)	35.2^b ^± 26.3 (0-100)	128.2^a ^± 29.5 (73-215)	4.9^a ^± 1.6 (2.1-7.8)
s IVP^y^	120	74.0^a ^± 11.6 (53.3-100)	30.8^b ^± 13.8 (0-50.0)	123.1^a ^± 28.9(65-188)	3.8^a ^± 2.7 (1-10.8)
dmapIVP^z^	143	72.8^a ^± 5.0 (67.3-76.9)	28.6^b ^± 3.9 (25.0-32.7)	124.2^a ^± 36.7 (65-175)	5.3^a ^± 2.8 (1.2-11.0)

Cleavage and blastocyst development rates were calculated based on total presumed zygotes transferred into IVC medium.

^a, b ^Values with different superscript letters within a column differ significantly (P < 0.01).

^x ^Development rates are those of OPU cultured zygotes. The development rates of agar-embedded slaughterhouse embryos were not considered.

^y ^A group of 10 COCs from slaughterhouse ovaries was used for IVP.

^z ^Slaughterhouse COCs pre-treated with 2 mM 6-dimethylaminopurine for 6~7 h before maturation followed by IVM, IVF and IVC.

### Blastomere numbers

The total number of blastomeres and the percentage of apoptotic blastomeres in the blastocysts did not differ (P > 0.05) between the controlOPU, agarOPU and sIVP groups (Table 2). Moreover, inhibition of maturation by DMAP did not affect the total number of blastomeres or the percentage of apoptotic blastomeres in the blastocysts (Table 2).

### Gene expression

**Figure 3:**
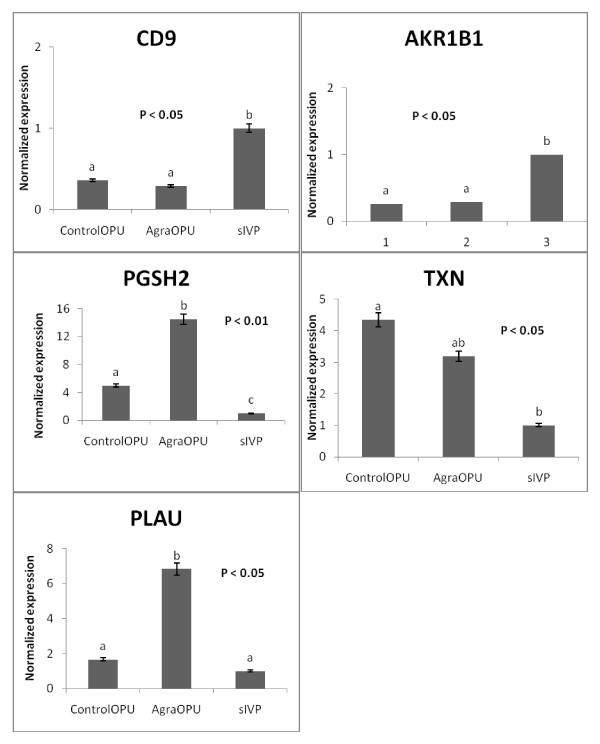
**Gene expression of day 8 bovine blastocysts**. Quantification of *CD*9, *AKR1B*1*, PGSH*2*, TXN *and *PLAU *genes in day 8 blastocysts developed from controlOPU (IVP with single cow presumed zygotes), agarOPU (IVP of single cow presumed zygotes with agar embedded slaughterhouse presumed zygotes) and sIVP (*in vitro *embryo production from slaughterhouse presumed zygotes) culture systems. In the agarOPU culture, only blastocysts developed from OPU presumed zygotes were used for gene expression analysis. Bars labeled with different letters differed significantly from each other.

The differential expression of selected genes was investigated by quantitative real time PCR. Expression was normalized to at least two reference genes. The expression of *CD9*, *AKR1B1*, *PGSH2*, *TXN *and *PLAU *in blastocysts derived from the controlOPU, agarOPU and sIVP culture systems is presented in Figure 3. The expression of *CD9 *and *AKR1B1 *was higher in sIVP blastocysts than in controlOPU and agarOPU blastocysts (Figure 3). By contrast, the expression of *PGSH2, ATP5A1 *and *PLAU *was higher in agarOPU blastocysts than in controlOPU and sIVP blastocysts (Figure 3). The expression of *TXN *was higher in OPU-derived blastocysts (controlOPU and agarOPU) than in sIVP blastocysts. However, the expression of *PLAC8 *(2.1- vs. 3.8- vs. 1.0-fold)*, TGFβ1 *(0.8- vs. 0.7- vs. 1.0-fold)*, ODC1 *(1.1- vs. 1.2- vs. 1.0-fold), *ATP5A1 *(0.8- vs. 1.1- vs. 1.0-fold) and *CASP3 *(0.8- vs. 0.8- vs. 1.0-fold) did not differ (P > 0.05) between the controlOPU, agarOPU and sIVP groups, respectively.

## Discussion

One of the challenges of the OPU-IVF technique is the scare of the COCs per culture droplet, particularly when small numbers of single cow COCs are used for *in vitro *embryo production. Recently Senatore et al. [11] developed the agar chip-embedded helper embryo coculture system for small numbers of OPU-derived presumed zygotes. The present study confirms that agar chip-embedded helper embryo coculture not only improves the blastocyst development rates of single cow OPU presumed zygotes but also maintains blastocyst quality as defined by blastomere numbers, the percentage of apoptotic blastomeres and blastocyst gene expression.

In the present study, 2~8 OPU-derived COCs were available for IVP per OPU session. Considering the limited number of COCs retrieved per cow, all of the presumed zygotes of a cow (2~8 per session) were used and a threshold of ten zygotes was reached per culture by adding agar chip-embedded slaughterhouse presumed zygotes (8~2). This variation in the number of COCs retrieved is consistent with other reports on cattle [1-3]. The number of COCs retrieved varies between individual donor cows [27-29]. Technical differences in OPU methods can also affect the number of COCs retrieved per OPU session [6,30,31]. In addition, variations in the number of COCs retrieved were observed between OPU sessions (data not shown). Therefore, it is difficult to culture the same number of OPU-derived presumed zygotes over the entire IVP process. The results of the present experiment are consistent with those of Senatore et al. [11] who cultured nine agar-embedded slaughterhouse presumed zygotes with one OPU zygote, seven with three OPU zygotes, and five with five OPU zygotes (i.e., nine zygotes added to one; seven added to three; five added to five) and compared the development rates of these cocultures to those of one, three and five OPU zygotes, respectively, each in a 50 μL droplet of medium. They reported that the development rate of OPU embryos cocultured with agar-embedded slaughterhouse helper embryos was superior to that of control embryos. The present study differs from that report in that the number of COCs collected during an OPU session from a single cow was considered as a unit of experimental replication. In addition, the present study assessed blastocyst quality by measuring blastomere numbers, the percentages of apoptotic blastomeres in the blastocysts and gene expression. The low development rate of OPU presumed zygotes in the controlOPU culture is not due to a compromised culture condition since 30.8% of the slaughterhouse presumed zygotes developed into blastocysts. Moreover, the agar does not affect the developmental competence of OPU embryos [11]. Therefore, the number of presumed zygotes per droplet may be the major determinant of compromised development in the controlOPU cultures.

Gopichandran and Leese [10] showed that, in cultures of multiple bovine embryos, the density and the distance between embryos determines the efficiency of embryo development. Moreover, the number of embryos per culture droplet is also important to embryo development [32-34]. The benefit of coculturing multiple embryos is likely due to the secretion of embryotrophic factors by the embryos during their development [10,35,36]. Several of these factors, including insulin-like growth factor (IGF)-I, IGF-II, platelet-activating factor [10,37] and platelet-derived growth factor [38], have been investigated. These growth factors have positive effects on IVP, particularly at low embryo density. However, the effect of these embryo-derived factors on the agarOPU culture system is unknown.

Blastocyst quality is measured by the total number of blastomeres and by the percentage of apoptotic blastomeres [23,25,39], which are inversely correlated with the post-transfer development potential of embryos [39-41]. The number of blastomeres and the percentage of apoptotic blastomeres in the blastocysts were evaluated by TUNEL assay. The level of apoptosis in the blastocysts was further confirmed by the detection of *CSAP3 *mRNA. No difference in the percentage of apoptotic blastomeres or *CASP3 *mRNA expression was detected among blastocysts from the three culture conditions. The number of blastomeres and the percentage of apoptotic blastomeres observed in blastocysts developed in the agarOPU, controlOPU and sIVP culture systems are consistent with published studies using OPU [7] and slaughterhouse [23] blastocysts. These results show that the agarOPU culture system has no adverse effect on the total number of blastomeres and does not promote blastomere apoptosis.

6-Dimethylaminopurine (DMAP), a serine threonine protein kinase inhibitor, can prevent the resumption of meiotic maturation in immature oocytes [42-44]. Slaughterhouse oocyte maturation was synchronized by pre-maturation exposure to DMAP for 6 to 7 h to coordinate the culture schedule of OPU-derived oocytes. The pre-treatment of oocytes with DMAP did not affect blastocyst rates, blastomere numbers or blastocyst apoptosis, which is inconsistent with previous studies of bovine oocytes [42-44]. Our experimental design differs from those studies in that immature oocytes were cultured with DMAP for a short period of time compared to the 12-24 h treatment used in those studies. Moreover, pre-treatment of human oocytes with DMAP for 7 h does not have any negative effect on embryo development [45]. These results show that the short-term pre-treatment of oocytes with DMAP has no effect on blastocyst development and quality. Therefore, the use of DMAP to synchronize the maturation of slaughterhouse COCs to the OPU schedule did not affect OPU embryo development in the agarOPU culture system.

*CD*9, a member of the tetraspanin family, might be involved in embryo invasive behaviors [46]. The *AKR1B*1 gene is potentially involved in pregnancy failure through metabolism of progesterone and synthesis of prostaglandin F_2α _(PGF_2_α) in the bovine uterine endometrium [17]. Aldose reductase induces apoptosis in cardiomyocytes [47]. High expression of these two genes in blastocysts correlates with the inhibition of embryo implantation and/or embryo resorption [20]. Therefore, down regulation of the *CD*9 and *AKR1B*1 genes in the agarOPU and controlOPU blastocysts might indicate improved embryo quality.

The *PGSH*2 gene is involved in the elongation and subsequent implantation process. Implantation and decidualization of the uterine stroma are impaired in *PGSH*2-null mice [48]. Mutation of the gene encoding *PGSH*2 in mice leads to infertility [48-50]. Moreover, embryo-derived prostaglandins mediate the interaction of embryos with the uterus during implantation in sheep [51]. *TXN *is also involved in the implantation process [52] and in the prevention of oxidative stress during *in vitro *embryo development [52,53]. *PLAU*, which is involved in implantation in humans [54], mice [55] and rats [56], is abundant in the biopsies of bovine blastocysts that resulted in calf delivery [20]. *PLAU *mRNA was up-regulated in agarOPU cultures compared to control cultures. Low expression of *PLAU *is associated with implantation failure in mouse embryos [57]. These results collectively suggest that the high expression of the *PGSH*2, *TXN *and *PLAU *genes in OPU blastocysts may be an indicator of high quality embryos.

*PLAC*8, an invasion-specific gene, is involved in placenta development and in the maintenance of the maternal-fetal interface. High expression of this gene in bovine blastocysts is associated with calf delivery [20]. Mouse embryos show a marked decrease in implantation when *TGF-B2 *is neutralized by the injection of neutralizing antibodies [58]. *TGF-B1 *favors trophoblast attachment and implantation by stimulating human cytotrophoblast cells to produce oncofetal fibronectin, which may serve as a trophoblast-uterine connecting protein [59]. *ODC1*-deficient murine embryos develop normally to the blastocyst stage and implant but die shortly thereafter [60]. Abundance of *ODC1 *in bovine blastocysts is associated with subsequent resorption of embryos [20]. *ATP5A1*, which binds ATP, is abundant in biopsies of bovine blastocysts that do not result in pregnancy. However, the levels of *PLAC8, TGF-β1, ODC1 *and *ATP5A1 *transcripts did not differ between the three groups.

Two important concerns are associated with the commercial application of the agarOPU culture method: the possibility of disease transmission and the difficulty of handling embryos. Preventing contamination of OPU blastocysts by agar-embedded slaughterhouse helper embryos is the major challenge of the culture system. Blastocysts produced from slaughterhouse ovaries are sometimes contaminated with viruses, bacteria and other pathogenic agents [61]. Interestingly, the zona pellucida prevents penetration of these agents into intact oocytes and embryos during the course of *in vitro *maturation and culture [62]. Therefore, washing the embryos before freezing and/or transfer, as is recommended by the International Embryo Transfer Society [63], can minimize disease transfer to recipient cows. Moreover, recipient cows transferred with experimentally BVDV-contaminated blastocysts were not infected with the virus [64]. Therefore, blastocysts produced by the agarOPU method may be used for embryo transfer without any serious risk of disease transmission. Moreover, the preparation of agar chip-embedded helper embryos requires extra precautions so that the slaughterhouse embryos remain isolated from the OPU embryos throughout the culture period.

## Conclusions

The present study demonstrated that the blastocyst development rates of small numbers of OPU-derived zygotes from a single cow can be improved by increasing the number of embryos per droplet through the coculture of slaughterhouse presumed zygotes with the OPU presumed zygotes. Moreover, the agarOPU coculture system has no adverse effect on embryo quality as measured by blastomere number, the percentage of apoptotic blastomeres in the blastocysts and gene expression. The agar chip-embedded helper embryo coculture system may be used in OPU-based IVP procedures to produce embryos of known genetic background from donor cows of high genetic merit.

## Competing interests

The authors declare that they have no competing interests.

## Authors' contributions

GKD performed embryo culture, embryo staining for the detection of total and apoptotic blastomere numbers, and qPCR analysis. JIJ performed OPU operations. THK, BHC and JIB managed donor cows and assisted with the OPU operations. SRD assisted with the *in vitro *embryo production from slaughterhouse ovaries and qPCR analysis. IRC arranged donor cows for OPU operations. IKK reviewed the manuscript and supervised all experimental activities related to this manuscript. All authors read and approved the final manuscript.
